# Individual and community-level factors associated with women’s utilization of postnatal care services in Uganda, 2016: a multilevel and spatial analysis

**DOI:** 10.1186/s12913-024-10636-6

**Published:** 2024-02-09

**Authors:** Moses Festo Towongo, Enock Ngome, Kannan Navaneetham, Gobopamang Letamo

**Affiliations:** Faculty of Social Sciences, Department of Population Studies, University of Botswana, Gaborone, Botswana

**Keywords:** Individual and community level factors, Postnatal care services utilization, Spatial analysis, Uganda

## Abstract

**Background:**

Over time, Uganda has experienced high levels of maternal mortality (435 deaths per 100,000 live births in 2006 to 336 deaths per 100,000 live births in 2016). The persistence of high levels of maternal mortality jeopardizes the achievement of Sustainable Development Goal (SDG) 3.1, which calls for reducing maternal mortality to 70 deaths per 100,000 live births by 2030. Conversely, the utilization of postnatal care (PNC) services in Uganda remained very low and has varied across regions. This study examined the individual and community-level factors influencing women’s utilization of postnatal care services in Uganda.

**Methods:**

Secondary data from the 2016 Uganda Demographic and Health Survey (UDHS) were used in this study. The study population consisted of women aged 15 to 49 who reported giving birth in the five years preceding the 2016 UDHS survey. The factors associated with postnatal care services were identified using multilevel binary logistic regression and spatial analysis.

**Results:**

The result shows that the prevalence of postnatal care service utilization in Uganda was low (58.3%) compared to the World Health Organization (WHO) target of 100%. The univariate analysis shows that 13.7% of women were adolescents, 79% were of higher parity, and 70.4% had primary/no formal education, of which 76.6% resided in rural areas. On the other hand, the multilevel analysis results showed that women aged 20-29 years and 30-39 years were also found to be more likely to use PNC services (AOR = 1.2, 95% CI: 1.01-1.47). Women who received quality ANC (AOR = 2.1, 95% CI: 1.78–2.36) were more likely to use postnatal care services than their counterparts. At the community level, women who lived in media-saturated communities were more likely to use postnatal care services (AOR = 1.3, 95% CI: 1.01–1.65). The spatial analysis found that the Central, Eastern, and Northern regions were the areas of hotspots in the utilization of postnatal care services.

**Conclusion:**

This study found that age, parity, level of education, place of residence, employment status, quality of the content of antenatal care, and community media saturation were the predictors of postnatal care service utilization. The spatial analysis showed that the spatial distributions of postnatal care service utilization were significantly varied across Uganda. The government must expand access to various forms of media throughout the country to increase PNC utilization.

## Introduction

In 2017, it was reported that 810 women died every day from pregnancy or childbirth complications worldwide. The majority of these deaths occurred in low-income countries, and they could have been avoided [1, 2]. Sub-Saharan Africa had the highest rate of maternal mortality (542 deaths per 100,000 live births) [3]. The persistence of maternal mortality jeopardizes the achievement of Sustainable Development Goal (SDG) 3.1, which calls for reducing maternal mortality to 70 deaths per 100,000 live births by 2030 [4]. According to evidence, most maternal deaths occur within the first 24 hours after delivery [5, 6]. Hemorrhage, hypertensive disorders, puerperal sepsis, and prolonged or obstructed labour are among the most common causes of maternal death in developing countries [7]. The World Health Organization (WHO) recommends that women receive postnatal care within the first two days after delivery to reduce maternal and infant mortality [6]. Because the majority of these deaths occur during the postpartum period, early utilization of postnatal care services is critical in managing and detecting complications [6, 8, 9]. Furthermore, early postnatal care services allow women to receive early education about healthy behaviours such as exclusive breastfeeding, nutrition, and effective family planning [10]. Despite the importance placed on postnatal care service utilization, disparities in Postnatal Care(PNC)service utilization persist across developing countries [11–13]. Besides, postnatal care service utilization studies are scarce in sub-Saharan Africa [14–16]. Further, postnatal care utilization varied regionally in sub-Saharan Africa, ranging from 73.51% in Central Africa to 31.71% in Eastern Africa [17].Similarly, these percentages varied according to specific countries, ranging from 26.6% in Eswatini to 94% in Burkina Faso [18]. Conversely, the percentage of women in Uganda who gave birth in healthcare facilities and received an immediate postnatal check increased from 35.7% in 2006 to 46.6% in 2011 and 65.0% in 2016 [19].However, the recent Uganda Demographic and Health Surveys (UDHS) report showed that only 54% of women who reported having given birth in the five years preceding the survey used postnatal care services within 48 hours as opposed to 100% WHO recommendations [19, 20]. Over the years 2006–2016, this has become one of the major contributors to maternal mortality in Uganda (435 deaths per 100,000 live births compared to 336 deaths per 100,000 live births in 2016) [20, 21].Therefore, to improve the state of maternal healthcare, Uganda, in her Health Sector Development Plan (UHSDP) 2015/16-2019/20, aimed to reduce maternal mortality to 320 deaths per 100,000 live births [22]. Conversely, by the end of UHSDP 2019/20, maternal mortality in Uganda had increased to 336 deaths per 100,000 live births [23, 24]. Previous studies have established the role of sociocultural, economic, and demographic factors in influencing the individual utilization of postnatal care services [18, 24–40]. Besides, recent postnatal care service utilization studies have increasingly used a multilevel approach to examine individual and community-level factors [17, 41–45]. Studies have revealed that postnatal care service utilization varies geographically [11, 46–50]. Factors such as region, level of education, wealth index, maternal age, number of ANC, place of delivery, place of residence, media exposure, and community illiteracy influence postnatal care service utilization.This varies from place to place and individual to community due to the influence of women’s culture and belief system [41, 43, 51–54]. Despite a large body of literature on spatial and multilevel analysis of postnatal care service utilization, such studies are uncommon in Uganda [55]. Previous research on postnatal care service utilization in Uganda using Uganda Demographic and Health Survey (UDHS) data has primarily focused on individual level analysis using ordinary regression, ignoring the multilevel nature of the data [19, 25, 56–58]. Also, studies in Uganda that used multilevel data analysis ignored spatial variation in postnatal care service utilization [55]. A spatial and multilevel study is critical for identifying high-risk geographical areas within a community and for designing community-based interventions in affected areas [43]. As a result, studies on the spatial analysis of postnatal care service utilization are scarce in Uganda because the geographic differences in postnatal care services are poorly documented. In addition, the use of multilevel analysis is critical because factors influencing postnatal care utilization operate at various levels. The use of techniques such as the ordinary regression model to analyze multilevel data has limitations because it assumes independence among observations within the clusters. Further, it has been argued that using standard binary regression models to regress factors at different levels leads to bias and a loss of power. Besides, aforementioned type of analysis may lead to an atomistic fallacy [43]. Evidence-based policy requires a thorough understanding of PNC in terms of spatial distribution and predictive factors. As a result, designing program interventions aimed at increasing postnatal care service coverage in Uganda necessitates knowledge based on spatial distributions and multilevel analysis. The purpose of this study was to look into the individual and community-level factors that influence women's use of postnatal care services in Uganda.

## Methods

### Study area

The Republic of Uganda is located in East Africa, on the equator. It is a landlocked country bordered on the east by Kenya, on the south by Tanzania, on the southwest by Rwanda, on the west by the Democratic Republic of Congo, and on the north by South Sudan. Its capital city is Kampala. The country covers area of 241,039 square kilometers and has 112 administrative districts [59]. It has a total population of 34,856,813 people, with 17,921,357 of them being women [60]. The country has a total of 6,937 health facilities (public, private, and non-profit) [61].

### Source of data and study sample size

The data came from the 2016 Uganda Demographic and Health Survey (UDHS). The data was retrieved from MEASURE DHS, which included the women’s standard file and a Geographic Information File (GPS) file containing survey cluster coordinates. The author requested the MEASURE DHS by briefly stating the objectives of this study, and access to the data was granted on the website https://dhsprogram.com. The study included a weighted sample of 9590 women aged 15 to 49 who gave birth in the five years preceding the survey in the selected Enumerated Areas (EAs). An EA is a natural village in rural areas and a city block in urban areas. The EAs in this study constitute a community or neighbourhood. More information about the study sample can be found in the 2016 UDHS report [20].

### Outcome variable

The outcome variable for this study is the utilization of postnatal care services. This variable was generated using the new World Health Organization (WHO) framework, which recommends that skilled health personnel check women’s health within 48 hours after delivery at the health facility or home delivery [6]. The outcome variable is coded into binary: “1” represents a woman whose health was checked by skilled health personnel within 48 hours after delivery, and “0” otherwise.

### Individual-level variables

Individual-level variables include women’s level of education, employment status, place of residence, religion, parity, maternal age, marital status, sex of the household head, family size, wealth index, and quality of ANC. Each of the variables was defined as follows: Education was defined as the highest level of education attained by the mother and categorized as no education, primary, secondary, and above. Employment was defined as a woman who reported to have worked during the past 12 months preceding the survey and was categorized as either unemployed or employed. Place of residence was defined as the type of dwelling where the woman resided, classified as rural or urban. Religion was defined as the woman’s religious affiliation and was categorized as Anglican, Catholic, Muslim, and other religious groups. Parity refers to the number of times a woman has delivered an infant of viable gestation or foetal weight, irrespective of the birth outcome [62]. Parity is strongly associated with postnatal care utilization; it was argued that women tend to pay more attention to their first pregnancy due to a lack of experience, and thus, they are more likely to use maternal health care services compared to their counterparts [63]. Maternal age at birth was categorized as 15–19, 20–24, 25–29, 30-34, and 35+ years. Marital status was defined as being single, married, living together, or previously married (separated, divorced, or widowed). The sex of the household head is defined as male or female recognized as the household head of the unit by members of the household or by him/her. Family size is defined as the number of family members in the household, which is divided into four categories: <4, 5-6, and 7+. The wealth index was a measure of household material wealth and was classified in this study as poor (comprised of the two lowest quintiles), middle, and rich (consisting of the two highest quintiles). The quality of antenatal care (ANC) was derived from whether the woman reported her weight, blood pressure, urine sample, and blood samples and whether she bought or was given iron tablets and fansidar for malaria during her antenatal care visits. A dichotomous variable was constructed from these responses. Those who used at least six ANC components were considered high quality, while those who used fewer than six components were deemed low quality.

### Community-level variables

The Uganda Demographic and Health Survey (UDHS) Enumerated Areas (EAs) were used as the unit of analysis to represent the community. The individual-level variables were aggregated into the EAs to represent community-level variables. This study contained four community-level variables, including community mean distance to the health facility, socioeconomic status, ethnicity diversity, and community media saturation, as explained below.

### Community mean distance to a health facility

The Community Mean distance to the health facility refers to the average distance of households to the nearest health facility in the community (Enumerated Areas). The geospatial datasets for Enumerated Areas (EAs) were obtained from UDHS, while those for health facilities were obtained from the Uganda Bureau of Statistics (UBOS). However, the UDHS localized GPS is not precise distance but random within the administrative entities. Go-located data were displaced to safeguard the privacy of respondents. The displacement procedure relocates the latitude and longitude according to a set of predetermined parameters viz: Urban locations were displaced 0-2 kilometres, rural locations 0-5 kilometres, and 1% (or every 100th point) are displaced 0-10 kilometres [64]. The distance to the health facilities was measured using ArcGIS software version 10.6.1 on weighted 649 Clusters whose coordinates were valid to the nearest 5, 229 health facilities II-IV, and the average distance to the nearest health facility was calculated using Euclidian distance. The near function in ArcGIS version 10.6.1 was used to compute the nearest health facilities within the EA catchment areas. Hence, the generated distances within the catchment areas of the EA were averaged and assigned to individual women in prospective EAs. Based on Uganda Health Sector Development Plan (UHSDP) I2000/01-2004/05, which recommended access to health facilities to be within a 5 km radius, a woman who resides in a household within an average distance of 5km radius was considered to have access to the health facility, otherwise inaccessible [22, 65].

### Community socioeconomic status

The community socioeconomic status measured the percentage of households in the wealth index’s lowest quintile [66]. The variable was constructed based on the household wealth index variable and defined as the proportion of women from poor and poorest households in the clusters. A woman was considered of low socioeconomic status if she was from a poor and poorest household; otherwise, she was considered of high socioeconomic status.

### Community ethnicity diversity

The community ethnicity diversity index is defined as the number of different ethnic groups and their proportional representation in the cluster. A formula of Simpson was adopted to generate ethnicity diversity, which captures the number of other groups in a community (cluster) and the relative representation of each group [67].

$$\mathrm{Ethnicity diversity index }=1-\sum_{{\text{i}}=1}^{{\text{n}}}{\left[\frac{{{\text{x}}}_{{\text{i}}}}{{\text{y}}}\right]}^{2}$$

Where: x_i_ = population of ethnic group i of the cluster, y= total population of the cluster, and *n*= number of ethnic groups in the cluster. The Simpson’s score ranges from 0.00 to 1.00. The 0.00 score symbilzes absence of diversity (homogeneity). While 1.00 score symbolizes absolute (perfect) diversity/heterogeneity. The cut-off point for heterogeneity (diverse) in this study ranges from 0.41-1.00 and homogeneity (less diverse) ranges from 0.00-0.40 [68].

### Community media saturation

Community media saturation is defined as the proportion of women who were exposed to one form of mass media information (radio, newspaper, television). Community media saturation is measured by the proportion of women who have been exposed to at least one media. Those with access to any form of media were regarded as high, and those without access to any form of media were regarded as low saturation. The three forms of media were summed up to create the index of exposure to media. The score ranged from 0 to 3, and 0 means no access, while 1-3 means a woman is exposed to at least one form of media. The aggregated variable is not normally distributed; the midpoint point of 50% was used as a cut-off point. Therefore, any proportion below 50% was recoded to “0” representing low saturation, and otherwise “1” representing high saturation [69, 70].

### Statistical methods

STATA V.14.2 software was used to analyze the data, and Microsoft Excel 10 was used for cleaning and coding for spatial analysis. On the other hand, ArcGIS V.10.6.1 was used for the spatial analysis and mapping. Sample weights were used to overcome the problems of geographical variability and non-response. The DHS framework for approximate-level weight reports [71] contains detailed information on the weighting procedure. After preparing the data for spatial analysis, it was imported into ArcGIS V.10.6.1, where the outcome variables were combined with secondary GPS data already projected from the DHS website (http://dhsprogram.com) [20] prior to analysis.

### Statistical analysis

Descriptive statistics such as frequency were computed and presented using tables. A bivariate analysis was conducted between the individual and community-level characteristics and postnatal care service utilization. The chi-square test examined the statistical association between outcome variables and covariates. The “svyset” module in the STATA software was used to account for the complex sample by taking into account the three pieces of design elements; weights, enumerated areas (EAs), and the strata [70]. The categorical variables in the bivariate model were tested for multicollinearity using the Variance Inflation Factor (VIF). Any variable with a VIF greater than 10 was removed from the model. The results showed the absence of multicollinearity (mean VIF=1.28, Min VIF=1.01, Max VIF=1.98). The variables related to PNC utilization (*p*<0.25) were thought to fit into a multivariate model. Since the 2016 UDHS data is hierarchical, and the outcome variable is dichotomous, a multilevel logistic regression modelling was found to be appropriate to test the association between postnatal care service utilization and women’s background characteristics. The enumerated areas (EAs) were used as a community; hence, the likelihood of women seeking PNC will likely be correlated with the EAs. The multilevel regression equation is stated as follows:

$$\mathit{log}\left[\frac{{\pi }_{ij}}{1-{\pi }_{ij}}\right]{=\beta }_{0}+{\beta }_{1}{x}_{1ij}+{\beta }_{2}{x}_{2ij}\dots +{\beta }_{n}{x}_{nij}+{uo}_{j}$$

Where *π*_*ij*=_ is the probability of the i^th^ individual in the j^th^ community utilization of postnatal care services. *(1-π*_*ij*_*)*= is the probability of the i^th^ individual in the j^th^ community who utilized postnatal care services late, β_0_ is the log odds of the intercept, β_1_, … β_n_ are the effect sizes of individual and community-level factors, X_1ij_... X_nij_ are independent variables of individual-level and community-level, u_Oj_, are the quantity of random errors at the cluster level. Four multilevel binary logistic regression models are employed to test the association between individual and contextual variables and the utilization of postnatal care services. No covariate was introduced in the first empty model (Model 1). The model is used to test the random effect of between-cluster variability. The interclass correlation coefficient (ICC) was estimated to establish if using the multilevel analysis method is justified by showing the level of variation between EAs [72]. The second model (Model 2) determined the effects of individual-level characteristics on women’s utilization of postnatal care services. The ICC was calculated and observed if there was any change in between-cluster variability upon adding the individual-level characteristics to the empty model. The third model (Model 3) introduced community-level characteristics and excluded the individual-level characteristics. In the fourth model (Model 4), which is the combined model, the individual and community-level characteristics were fitted to show their net fixed and random effects. The random effect was explained using the Interclass Correlation (ICC) using the following formula [ICC = σu^2^ / (σu^2^ + π^2^ /3)]. The fixed-effect size of individual and community-level factors on the utilization of postnatal care services was stated using the Adjusted Odds Ratio (AOR) and was estimated using 95% Confidence Intervals and *p*-values less than 5% [72, 73]. The log-likelihood ratio was used to test how adequate the model is, and Akaike Information Criteria (AIC) was used to assess how well the different models fitted the data [70].

### Spatial analysis

The spatial analysis was done using ArcGIS V.10.6.1. The global spatial autocorrelation was examined using Global Moran's I statistics (Moran's I) to determine whether the pattern was clustered, scattered, or random over the study area [74]. Moran's Index is standardized into a Z-score. A positive Moran's I Index value combined with a positive Z-score (>1.96, *p*-value 0.05) indicates a clustered pattern of PNC (hotspots/clustering), whereas a negative Moran's I Index value combined with a negative Z-score (< -1.96, *P*-value 1.96). A *p*-value of 0.05 indicates a dispersed pattern of PNC (cold spots/dispersion areas). GetisOrd Gi* statistics were used to identify postnatal care service utilization hotspots. The spatial distributions of postnatal care service utilization in unsampled areas were estimated or predicted using empirical Bayesian kriging interpolation [75].In positive global spatial autocorrelation, local clusters with high or low postnatal care service utilization in Uganda were identified using spatial scan statistics (SaTScan V.10.1 software) [76]. The Bernoulli probability model was used to detect women who utilized postnatal care services, and such women were considered cases, while women who did not utilize postnatal care services were considered controls. Based on 999 Monte Carlo replications, the most probable cluster was identified using the *p*-value and likelihood ratio test [69].

## Results

### Individual and community-level characteristics of the respondents

Table 1 shows the percentage distribution of women and their characteristics who reported having given birth in the five years preceding the surveys. Over half (53.32%) of the women were aged 20-29, while 13.68% were aged 19 years and below. Only a small proportion (5.34%) were over the age of 40 years. Over three-quarters of the respondents (81.24%) were married or living together. Meanwhile, the single women formed the lowest proportion (5.80%). The majority of respondents (73.10%) live in male-headed households. More than three-quarters (79.80%) of the women had two or more children. Over half (60.00%) of the women completed primary school, and 10.42% had no education. Additionally, more than a quarter (29.58%) of women had secondary or higher education. Above three-quarters (76.62%) of respondents live in rural areas. Over a quarter (41.35%) of the respondents fall into the poor category of the wealth index. Anglican and Catholic are the two largest religious groups among respondents (70.60%). A sizable proportion of respondents (79.08%) were employed. Only a small percentage (26.11%) of respondents reported receiving low-quality antenatal care content. A low proportion (27.12%) of respondents live in communities with high access to healthcare facilities. A sizable proportion of respondents (79.61%) belong to less diverse communities. Meanwhile, more than half (51.54%) of respondents lived in less media-saturated communities.

**Table 1:** Shows percentage of women who utilized of Postnatal Care Services by individual and household characteristics and the level of statistical associations

**Variables**	**Number (weighted N, %)N=9590**	**Not used PNC** **(Weighted %)**	**Used PNC** **(Weighted %)**	***P-*value**
**INDIVIDUAL VARIABLES**				
**Age at last birth**				**0.029**
<=19	1305 (13.68)	40.80	59.20	
20-29	5074 (53.32)	40.49	59.51	
30-39	2695 (27.65)	43.85	56.15	
40+	516 (5.34)	45.81	54.19	
**Marital Status**				**0.003**
Single	552 (5.80)	34.47	65.53	
Married	4078(40.44)	40.57	59.43	
Living together	3793 (40.84)	43.22	56.78	
Previous married	1167 (12.92)	44.00	56.00	
**Household Head**				**0.014**
Male	7006 (73.10)	42.62	57.38	
Female	2584 (26.90)	39.36	60.64	
**Parity**				**0.000**
1	1882(20.20)	33.37	66.63	
2-3	3195 (33.88)	40.00	60.00	
4+	4513(45.92)	46.71	53.29	
**Family Size**				**0.006**
<=4	3105 (33.36)	39.11	60.89	
5-6	2986(30.95)	42.53	57.47	
7+	3499 (35.69)	43.53	56.47	
**Level of Education**				**0.000**
No education	1208 (10.42)	47.39	52.61	
Primary	5869 (60.00)	47.57	52.43	
Secondary or Higher	2513 (29.58)	27.94	72.06	
**Place of Residence**				**0.000**
Urban	1985(23.38)	29.33	70.67	
Rural	7605 (76.62)	45.53	54.47	
**Wealth Index**				**0.000**
Poor	4494 (41.35)	47.99	52.01	
Middle	1773(18.84)	47.49	52.51	
Rich	3323 (39.82)	32.54	67.46	
**Religion**				**0.000**
Anglican	3034 (31.17)	44.60	55.40	
Catholic	3975(39.43)	41.47	58.53	
Muslim	1099(13.77)	33.68	66.32	
Other’s	1482 (15.63)	43.86	56.14	
**Employment Status**				0.220
Unemployed	1893 (20.92)	43.44	56.56	
Employed	7697 (79.08)	41.30	58.70	
**Quality of ANC content**				**0.000**
Low	7150 (73.89)	47.24	52.76	
High	2440 (26.11)	26.19	73.81	
**COMMUNITY VARIABLES**				
**Community Distance to Health Facility**				0.400
Inaccessible	6615(72.12)	48.66	51.34	
Accessible	2829 (27.87)	32.05	67.95	
**Community Socioeconomic Status**				**0.000**
Low	5496 (50.39)	47.00	53.00	
High	4094(49.61)	36.41	63.59	
**Community Ethnicity Diversity Index**				0.709
Less Diverse	7535(79.61)	43.34	56.66	
More Diverse	2055 (20.39)	39.64	60.36	
**Community Media Saturation**				**0.000**
Less Saturated	5321(51.54)	48.28	51.72	
Saturated	4269(48.46)	35.09	64.91	
**Total**	**9590 (100)**	**41.74**	**58.26**	

### Prevalence of postnatal care service utilization across the explanatory variables

Table 1 shows that 58.3% of women used postnatal care (PNC) services during their most recent birth. Individual/household-level characteristics such as age at last birth, marital status, sex of the household head, parity, family size, level of education, place of residence, wealth index, and religion were associated with the utilization of postnatal care services. Similarly, community-level characteristics such as community socioeconomic status and media saturation were significantly associated with using postnatal care services. In terms of age, women aged 20–29 had the highest proportion of postnatal care use (59.51%). This was followed by 59.20% of women aged less than 19. The proportion was lower for women aged 30-39 and over 40 (56.15% and 54.19%, respectively). Compared to married, living together, and previously married women (59.43%, 56.78%, and 56.0%, respectively), a significant proportion (65.53%) of single women used PNC services. Female-headed households used PNC services at 60.64%, while male-headed households used PNC services at 57.38%. Women who had four or more births (53.29%) used PNC services less than those who had one birth (66.63%). A slightly higher proportion (60.89%) of women from families with four members or less utilized PNC compared to those from large families (7+ members) 56.47%. A higher proportion of women with a secondary or higher level of education (72.06%) used PNC services compared to those with primary or no education (52.43% and 52.61%, respectively). The proportion of women using PNC in rural areas is lower (54.47%) than those residing in urban areas (70.67%). Women in the rich category of the wealth index used PNC services at a rate of 67.46%, compared to 52.51% and 52.01% in the middle and poor categories, respectively. Regarding religion, 66.32% of Muslim women used PNC services, compared to 55.40% of Anglicans, 58.53% of Catholics, and 56.14% of other religious groups. Women who reported being employed used PNC services at a higher rate (58.70%) than their counterparts (56.56%). Compared to women who received low-quality ANC content (52.76%), a higher proportion (73.81%) of women who received high-quality ANC content used postnatal care services. Furthermore, when compared to their counterparts, a higher proportion of women living in communities closest to a health facility, ethnically diverse communities, communities with high socioeconomic status, and communities with high media saturation used postnatal care services (67.95%, 60.36%, 63.59%, and 64.91%, respectively) (Table 1).

### Factors associated with PNC utilization among women in Uganda

#### Random effects (measures of variation)

The random effect or the community-level variation in the utilization of postnatal care services was assessed by interclass correlation coefficient (ICC), and model fitness was assessed using Akaike information criteria (AIC). The ICC of the null model (model) showed that 22% of the variation in the utilization of postnatal care services was associated with community-level factors. After adjusting for community and individual-level factors, the full model showed statistically significant variation in postnatal care utilization across communities at a 5% significance level. From the empty model, the ICC dropped to 19%. Therefore, the postnatal care service utilization clustering was related to individual and community-level characteristics. The AIC values confirmed a subsequent decrease, significantly improving from the empty model to the individual and community-level models. This confirms the goodness of fit of the final model found in this analysis. Therefore, model 4 was chosen to predict postnatal care services utilization among women in Uganda (Table 2).

**Table 2:** Multilevel analysis of odd ratios on the effect of individual, household and community level factors in influencing the utilization of postnatal care among women in Uganda, UDHS 2016

**Variables**	**Model 1**	**Model 2**	**Model 3**	**Model 4**
	**Empty Model**	**Individual level**	**Community level**	**Individual/Community level**
	**COR (95% CI)**	**COR (95% CI)**	**COR (95% CI)**	**AOR (95% CI)**
**INDIVIDUAL VARIABLES**				
**Age at last birth**				
<=19		1.00		1.00
20-29		1.18 (0. 97-1.43)		1.22*(1.01-1.47)
30-39		1.31* (1.04-1.67)		1.39**(1.10-1.75)
40+		1.21 (0.88-1.67)		1.29(0.94-1.77)
**Marital Status**				
Single		1.00		
Married		1.10 (0.83-1.44)		
Living together		1.05 (0.80-1.37)		
Previous married		0.93 (0.69-1.24)		
**Household Head**				
Male		1.00		
Female		1.02(0.90-1.17)		
**Parity**				
1		1.00		1.00
2-3		0.69***(0.57-0.84)		0.70*** (0.58-0.85)
4+		0.59*** (0.47-0.73)		0.58*** (0.47- 0.72)
**Family Size**				
<=4		1.00		1.00
5-6		1.05(0.91-1.21)		1.04 (0.90-1.19)
7+		1.04(0.89-1.21)		1.01 (0.87-1.17)
**Level of Education**				
No education		1.00		1.00
Primary		0.98(0.82-1.18)		1.00(0.84-1.20)
Secondary or Higher		1.55*** (1.25-1.91)		1.66***(1.34-2.05)
**Place of Residence**				
Urban		1.00		1.00
Rural		0.70**(0.56-0.87)		0.69***(0.54-0.90)
**Wealth Index**				
Poor		1.00		1.00
Middle		1.18 (0.99-1.41)		1.19* (1.01-1.42)
Rich		1.29** (1.09-1.52)		1.32**(1.12-2.56)
**Religion**				
Anglican		1.00		1.00
Catholic		1.06 (0.92-1.21)		1.06 (0.92-1.21)
Muslim		1.19 (0.95-1.50)		1.23 (0.97-1.55)
Other’s		0.99 (0.84-1.17)		0.99 (0.84-1.16)
**Employment Status**				
Unemployed		1.00		1.00
Employed		1.29** (1.11-1.50)		1.32***(1.34-1.54)
**Quality of content of ANC**				
Low		1.00		1.00
High		2.04***(1.81-2.49)		2.05***(1.78-2.36)
**COMMUNITY VARIABLES**				
**Community Socioeconomic status**				
Advantaged			1.00	1.00
Disadvantaged			1.39**(1.09-1.78)	0.92 (0.71-1.19)
**Community Media Saturation**				
Less Saturated			1.00	1.00
Saturated			1.58*** (1.24-2.02)	1.29*(1.01-1.65)
Random effect results:				
PSU Variance (95% CI)	0.95(0.78-1.15)	1.96(0.63-0.92)	0.84(0.69-1.02)	0.76(0.63-0.92)
ICC	0.22	0.19	0.20	0.19
Wild chi-square and p-value	Ref	χ^2^=262.37, *p*<0.000	χ^2^= 40.85, *p*<0.000	χ^2^=208.12, *p*<0.000
Model fitness:				
Log-likelihood	-2254979	-2182476.9	-2250233.4	-2210640.5
AIC	4509962	4365000	4500475	4421321
PSU	649	649	649	649
N	9,590	9,590	9,590	9,590

#### Measure of association (fixed effects) results

The individual/household and community-level variables were selected using enter methods 0.05 significant level. In model 4 in Table 2, which is the final model, the individual and community-level variables such as age at last birth, parity, level of education, place of residence, wealth index, employment, quality of content of ANC, and community media saturation were statistically significant at *p*<0.05. Women aged 30-39 and 20-29 years had higher adjusted odds of using postnatal care services than women aged 19 years (AOR = 1.4, 95% CI: 1.10-1.75, and AOR = 1.2, 95% CI: 1.01-1.47, respectively). The odds of using postnatal care services decreased with parity as women with two or more births were less likely to use postnatal care services (AOR = 0.7, 95% CI = 0.58–0.85 and OR = 0.6, 95% CI = 0.47–0.72, respectively). Women in rural areas were less likely to use postnatal care services than those in urban areas (AOR = 0.7, 95% CI: 0.54-0.90). Women with secondary or higher education were more likely to use postnatal care services than those with primary or no education (AOR = 1.7, 95% CI: 1.34–2.05). Women in the rich and middle wealth index were more likely than those in the poor wealth index to use postnatal care services (AOR = 1.3, 95% CI: 1.12-2.56, and AOR = 1.2, 95% CI: 1.01-1.42, respectively). Women who were employed were more likely than those who were not to use postnatal care services (AOR = 1.3, 95% CI: 1.34–1.54). Women who received high-quality ANC were more likely to use postnatal care services than those who received low-quality ANC (AOR = 2.1, 95% CI: 1.78-2.36). Furthermore, living in media-saturated communities increased the likelihood of women utilizing postnatal care services (AOR = 1.3, 95% CI: 1.01–1.65) (Table 2).

### Spatial analysis

#### Spatial autocorrelation

**Fig. 1:**
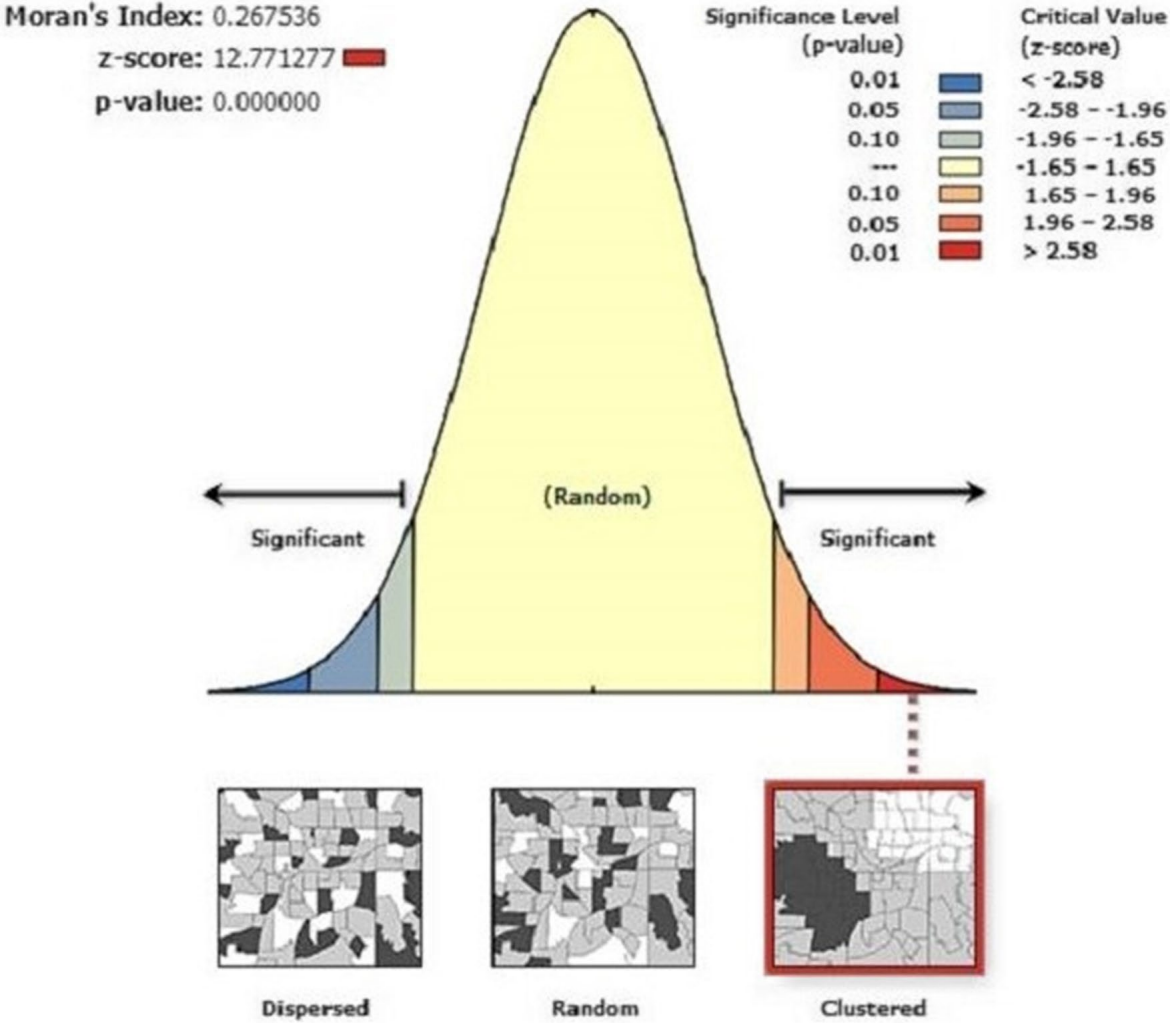
Spatial autocorrelation for postnatal care service utilization in Uganda, 2016

The spatial autocorrelation result indicates whether postnatal care service utilization in Uganda is random, dispersed, or clustered. It is worth noting that the spatial autocorrelation analysis in Uganda reveals that the postnatal care service utilization had a clustering effect in Uganda (Global Moran's I = 0.27, *p*-value 0.000). This means some areas have high utilization of postnatal care services, while others have low utilization. The output has generated a key on the left and right sides of each panel. A Moran's I index value closer to +1 indicates clustering, 0 indicates randomness and a value closer to -1 indicates dispersion in the utilization of postnatal care services among women in Uganda [77]. A statistically significant Moran's I (*p*<0.05) demonstrates the presence of considerable spatial autocorrelation/spatial dependency and leads to the rejection of the null hypothesis (postnatal care service utilization is randomly distributed). Figure 1 shows that the observed value is greater than the expected value, and the *p*< 0.05 indicates spatial variability in the utilization of postnatal care services among Ugandan women (Fig. 1).

#### Hotspot analysis of postnatal care service utilization

**Fig. 2:**
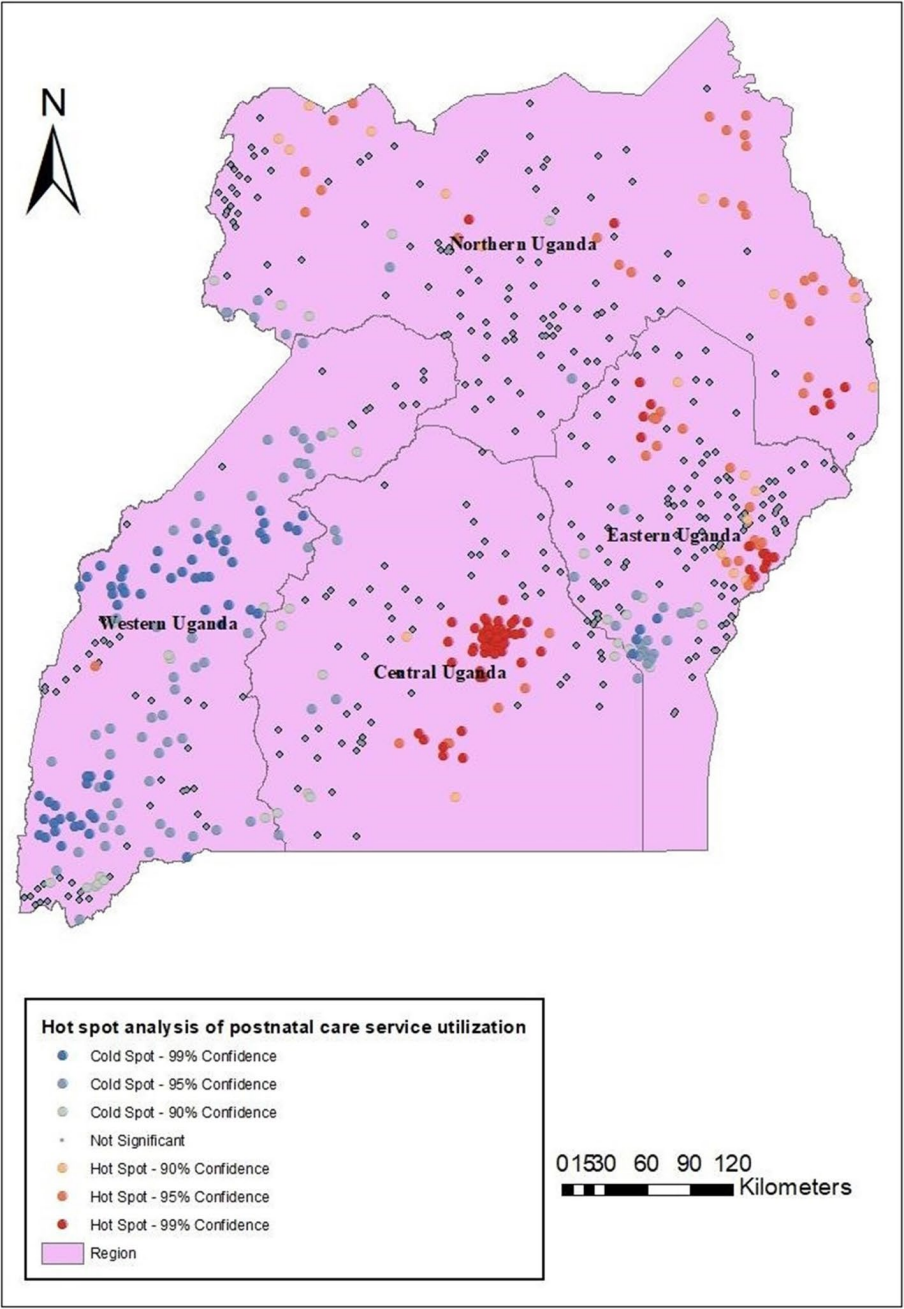
Shows the Hotspot Analysis for postnatal care service utilization in Uganda, 2016

The Getis-Ord GI* analysis was used to detect hotspots and coldspots for postnatal care service utilization in Uganda. Most of the hotspot areas, those with high prevalence rates of postnatal care service utilization, were in Uganda’s Central, Eastern, and Northern regions. In contrast, in most coldspots areas, those with a low prevalence rate of postnatal care service utilization were detected in the Western region (Fig. 2).

#### Interpolation of postnatal care service utilization

**Fig. 3:**
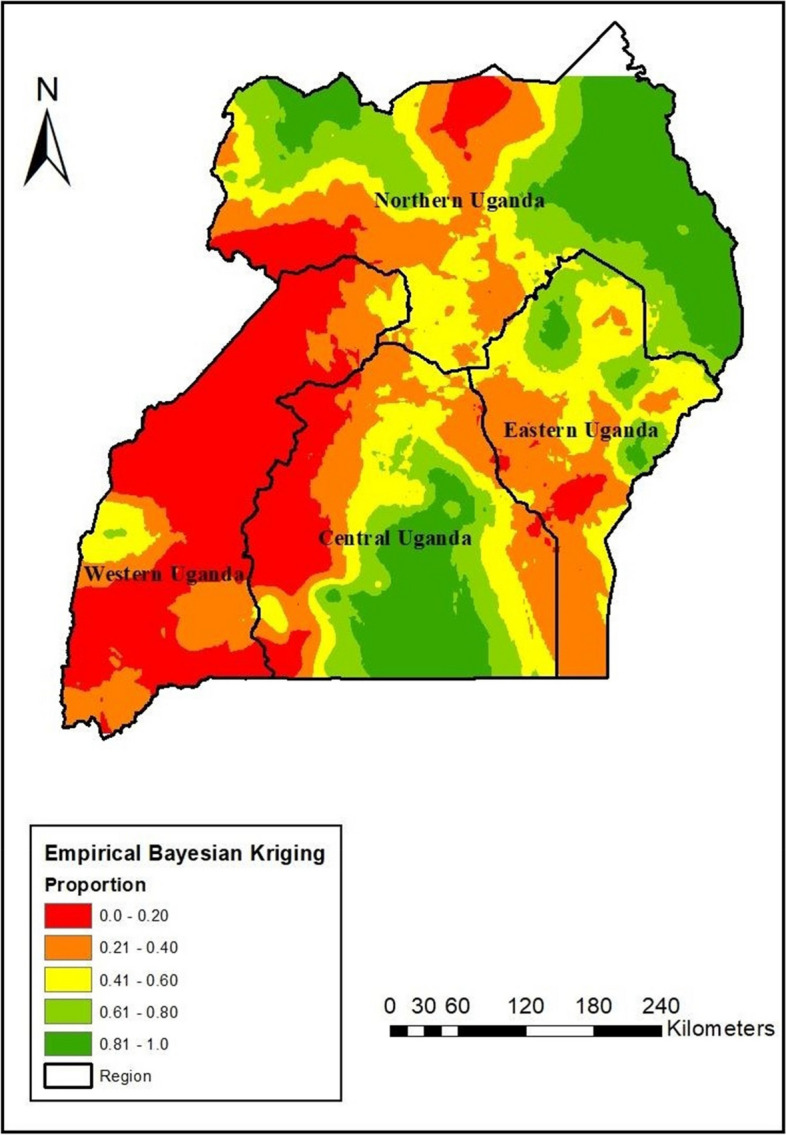
Empirical Bayesian Kriging for postnatal care service utilization among women in Uganda, 2016

Empirical Bayesian kriging interpolation analysis showed that the postnatal care service utilization was relatively higher in Central, Eastern, and Northern Uganda. Meanwhile, the low postnatal care service utilization region was mainly found in the Western region of Uganda (Fig. 3).

#### SaTScan analysis of postnatal care services utilization

**Fig. 4:**
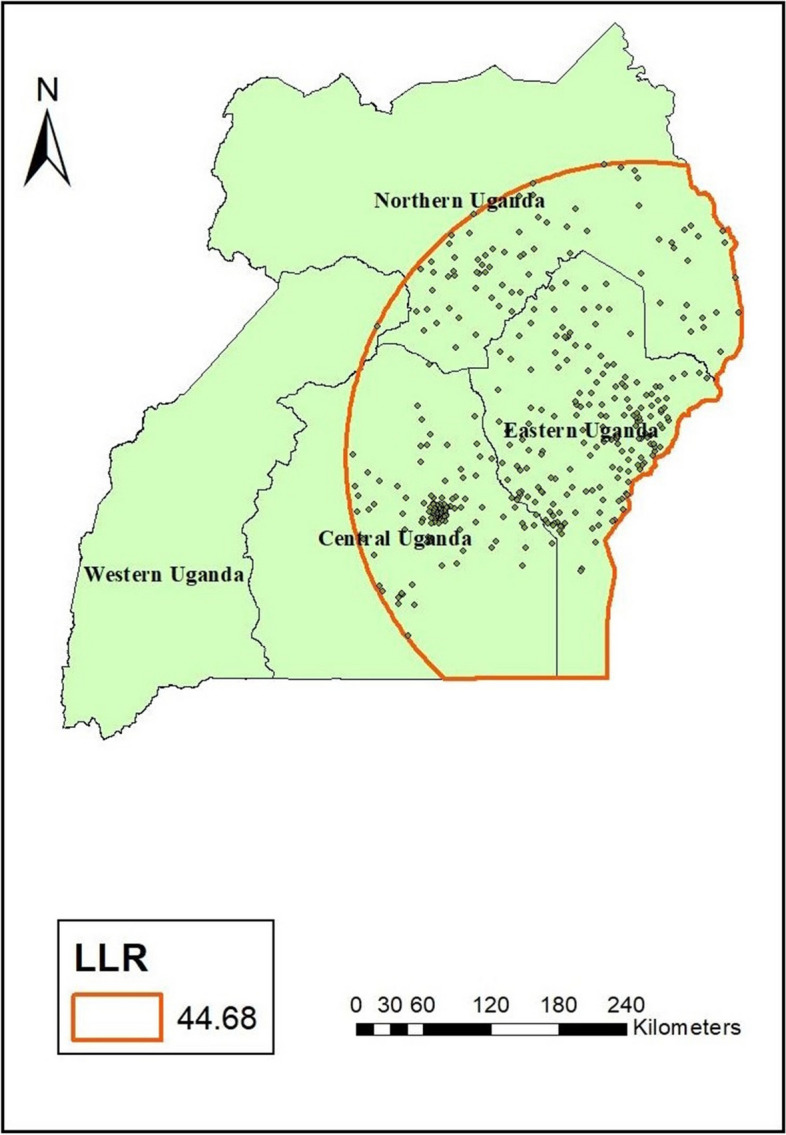
Spatial scan statistical analysis of hotspot areas of postnatal care service utilization in Uganda, 2016

The spatial scan statistics analysis for clusters with high rates was applied using the Bernoulli model on postnatal care service utilization in Uganda. A total of 384 were significant primary clusters of postnatal care service utilization. The primary clusters were found in Central, Eastern, and Northern regions of Uganda at 0.765256 N, 34.198923 E) at a radius of 261.49 km, with a log-likelihood ratio (LLR) of 44.7at a *p*-value 0.000. This means that women who reside in this spatial window are 44.7 times more likely to utilize postnatal care services compared to those outside this window (Table 3). The highest postnatal care service utilization cluster was observed in Uganda's Central, Eastern, and Northern regions (Fig. 4).

**Table 3:** The most SaTScan Clusters of Areas with Significant PNC Service Utilization, Uganda, 2016

EA (enumeration Area)	Regions	Coordinate or Radius	RR	LLR	*P*-value
90, 188, 191, 219, 189, 237, 218, 193, 231, 232, 234, 233, 225, 228, 197, 198, 235, 236, 258, 194, 224, 196, 184, 195, 200, 192, 201, 226, 220, 223, 227, 238, 257, 239, 217, 202, 256, 216, 255, 222, 229, 254, 215, 199, 185, 240, 241, 214, 247, 187, 230, 173, 206, 176, 245, 246, 213, 242, 182, 145, 203, 149, 244, 174, 262, 259, 205, 248, 243, 151, 263, 208, 186, 175, 183, 250, 152, 261, 249, 207, 264, 266, 150, 297, 204, 221, 144, 212, 265, 153, 209, 260, 665, 211, 670, 267, 155, 210, 268, 177, 251, 668, 171, 269, 298, 156, 148, 147, 154, 272, 678, 252, 253, 672, 673, 669, 676, 679, 674, 677, 146, 681, 271, 680, 172, 685, 682, 161, 683, 675, 162, 671, 168, 658, 165, 684, 169, 160, 276, 277, 270, 660, 294, 167, 158, 178, 667, 157, 663, 666, 283, 180, 179, 308, 164, 142, 143, 657, 275, 303, 159, 664, 181, 166, 291, 274, 299, 300, 281, 282, 331, 301, 163, 656, 279, 123, 131, 295, 280, 304, 288, 310, 289, 170, 306, 662, 124, 273, 302, 122, 128, 141, 659, 121, 278, 284, 287, 307, 661, 285, 296, 126, 292, 129, 127, 130, 286, 311, 138, 125, 293, 290, 140, 139, 118, 378, 655, 89, 117, 91, 92, 654, 90, 305, 79, 43, 309, 40, 44, 42, 371, 26, 372, 27, 39, 41, 38, 369, 45, 5, 9, 2, 370, 37, 10, 36, 30, 29, 32, 28, 7, 13, 35, 6, 33, 119, 80, 12, 1, 34, 8, 98, 4, 23, 96, 312, 31, 11, 652, 3, 97, 17, 24, 88, 99, 341, 25, 16, 20, 137, 94, 14, 21, 18, 19, 373, 15, 93, 336, 22, 77, 313, 95, 314, 344, 78, 74, 120, 102, 343, 132, 87, 653, 73, 71, 76, 332, 75, 364, 100, 72, 318, 101, 316, 330, 337, 374, 339, 350, 317, 70, 315, 342, 329, 362, 346, 363, 375, 69, 86, 338, 85, 376, 347, 365, 334, 379, 348, 340, 335, 353, 328, 352, 366, 380, 646, 116, 377, 351, 367, 368, 136, 645, 105, 349, 650, 651, 135, 358, 326, 641, 345, 642, 410, 68, 113, 355, 356, 333, 115, 381, 106, 67, 114, 354, 643, 327, 644, 361, 647, 465, 383, 392, 325	Central, Eastern and Northern Uganda.	(0.765256 N, 34.198923 E) / 261.49 km	2.39	44.68	0.000

## Discussion

Maternal mortality is unexpectedly high in Uganda, and it declined at a snail’s pace [20]; the utilization of postnatal care services within 48 hours is essential to improving the survival of both the mothers and newborns. Lack of proper care within 48 hours leads to complications for mothers and newborns [78]. Conversely, utilization of maternal healthcare services has varied across Uganda's geographical space [2, 20]. Therefore, this study investigated the individual and community-level factors associated with postnatal care service utilization among Ugandan women. The overall prevalence of postnatal care service utilization in Uganda was 58.3%, which is slightly higher than the findings by Ndugga [25]; the study has also shown variations regarding the utilization of postnatal care services across sub-Saharan African countries [17]. For instance, a higher proportion of women from Lesotho (81.5%), Cameroon (85.5%), Zimbabwe (84.2%) and Burkina Faso (81.6%) utilized postnatal care services compared to women in Uganda [17]. Conversely, a lower proportion of women from Swaziland (20.4%), Chad (48%), Ethiopia (8.3%), and Benin (19.1%) utilized postnatal care services compared to women from Uganda [17]. The plausible explanation for such variations in the utilization of postnatal care services between women from Uganda and other countries in sub-Saharan Africa could be explained by different health policies and sociocultural variations among these countries [17, 25]. Furthermore, the multilevel analysis showed that at the individual level, maternal age at last birth, parity, education, place of residence, wealth index, employment status, and quality of content of antenatal care services were the predictors that influenced the utilization of postnatal care services. Women aged 20–29 and 30-39 years were more likely to utilize postnatal care services compared to those aged ≤19 years. This study’s findings align with other studies from sub-Saharan African countries [17, 79]. The plausible explanation might be that as maternal age increases, women become more experienced regarding their physiology and how to manage complications arising after childbirth [17]. Therefore, programs encouraging teens and older women to utilize postnatal care services in Uganda could be crucial.

Further, in Uganda, among individual or household-level factors, women of higher parity were less likely to utilize postnatal care services than those of lower parity. The findings from this study are consistent with other studies [34, 57]. However, this relationship is not systematic in a study conducted by Ndugga (2020) in Uganda, who established no association between parity and early utilization of postnatal care services among women [25]. Hence, it is imperative to acknowledge that existing research has demonstrated a negative correlation between higher parity among women and their use of postnatal care services. The explanations ascribed to this phenomenon may be traced to the experiences individuals acquired throughout their initial childbirth experiences [34, 57].

Furthermore, women with secondary or higher education were more likely to utilize postnatal care services compared to those with primary or no education. This finding is consistent with another study by Dahiru [46]. The plausible explantion is that, educated women can make better health decisions and are more likely to have the financial means to pay for health care, so they do not have to rely on their husbands to seek care [2, 25]. Therefore, the government of Uganda is implored to invest more in education for all, with more affirmative action on girl child education as a long-term strategy to improve the utilization of postnatal care services.

Place of residence is associated with early utilization of postnatal care services. Women who reside in rural areas were less likely to utilize postnatal care services compared to those who live in urban areas. This finding is consistent with a study in Ethiopia [35]. Further, rural women are more influenced by traditional or cultural practices related to childbirth and postnatal care services than city women [17, 57]. However, not all city women are also entitled to easy access as perceived because inequality regarding the utilization of maternal healthcare services has long been documented to have existed among the urban poor in the urban slums [37].

The high wealth index bracket is also associated with postnatal care services. Women in the middle and upper wealth index were more likely than those in the lower wealth index to use postnatal care services. This study’s findings are consistent with those of other studies [46, 57]. One plausible explanation could be that higher-income women have more purchasing power and are more likely to meet the direct and indirect costs of healthcare services [37, 48]. Furthermore, they can access more information on postnatal care services than the poor [37, 48, 57].

Similarly, women’s employment status was also associated with using postnatal care services. Women employed were more likely to utilize postnatal care services than their counterparts. This finding corroborates with other studies in Ghana, Nigeria, and Uganda [42]. The primary reasons could be the ability to afford the cost associated with the direct and indirect private healthcare services at the health facilities [57]. Likewise, in a study conducted in Eastern Uganda, the scholars found that women employed in the formal sector were four times more likely to utilize early postnatal care services than those who were self-employed [38].

It is worth mentioning that the use of postnatal care services is related to the quality of antenatal care services. Women who received high-quality ANC content were more likely to use postnatal care services than those who received low-quality ANC content. This study’s findings are consistent with those of other studies [34, 79]. During ANC services, women may develop a rapport with health personnel, who may provide positive information, encouraging them to dispel myths about giving birth in a health facility and eventually receiving early postnatal care services [80]. Women are also discouraged from seeking care in health facilities due to dissatisfaction with the quality of ANC content. Additionally, this is exacerbated by service providers’ poor attitudes during antenatal care visits. This is because women who encounter mistreatment from health facilities are discouraged from using health facilities for birthing, resulting in late utilization of postnatal care services [58].

Community media saturation was linked to postnatal care services at the community level. Women who lived in heavily media-saturated communities were more likely to use postnatal care services than their counterparts. This study’s findings support other studies [17, 25, 79]. The education women received due to media access may have created awareness about the importance of PNC services and the complications that can arise during the postpartum period [17, 57].

Furthermore, the spatial distribution of postnatal care service utilization significantly varied across Uganda. The study identified postnatal care service utilization hotspots in Central, Eastern, and Northern Uganda. At the same time, the cold spots were observed in the Western region of Uganda. The empirical Bayesian kriging interpolation showed that there would be suitable utilization of postnatal care services in Central, Eastern, and Northern Uganda. Consistently, SaTscan analysis showed that the primary clusters were located in Central, Eastern, and Northern regions. Women who reside in primary clusters were more likely to utilize postnatal care services. This finding is consistent with other studies from Ethiopia [48, 50] that found variation in postnatal care service utilization. Similarly, these findings are consistent with other studies from Uganda, which established variations in the utilization of maternal healthcare services [2, 57]. The plausible reasons could be linked to the distribution of health facility infrastructure, accessibility, sociocultural milieu, and level of economic development, which are the main factors associated with regional differences in postnatal care utilization [41, 51].

Similarly, in Uganda, women in the Central region were more likely to utilize postnatal care services than others because the region hosts Uganda’s commercial and administrative headquarters [2]. Therefore, this region is well-serviced with social amenities such as improved road networks, health infrastructure, schools, and access to mass media compared to other regions. These factors are the engine for socioeconomic development, promoting access to health facilities and using maternal healthcare services [54]. In contrast, the Eastern region of Uganda is underdeveloped [54], and the sociocultural influence seems to be eminent in this region [57]. However, the results of this study show that women from the clusters in this region were more likely to utilize postnatal care services than women in the Western region. The plausible explanation could be attributed to underdevelopment, which tends to attract more development partners to support social services such as maternal healthcare [2]. Compared to the Eastern region, the Northern region is underdeveloped compared to the Western region [54], but the region depicted a higher usage of postnatal care services. This result was not expected, and it can be argued that, due to the conflict in the Northern region of Uganda, the government and other stakeholders could have shifted attention to alleviating suffering and reconstruction of the ravaged Northern region. Therefore, these initiatives might bring positive results of healthcare services closer to people, more especially in the internally displaced camps [40]. Conversely, women from the Western region were less likely to utilize postnatal care services than those from other regions. The finding was unexpected because the region is well-developed compared to the Eastern and Northern regions [54]. The plausible explanation could be this region's sociocultural influence on pregnancy and childbirth [81]. For example, culturally, children born at home are not supposed to be seen until after seven days, meanwhile those born normally at a health facility have to be rushed home to meet the cultural demand and, this tends to compromise the health status of the mother and baby to be checked within 48 hours [2, 81].

## Strength and limitations

The study’s strength stems from its large sample size and country-representative data. This study’s use of advanced statistical methods, such as geospatial and multilevel analysis, which accounted for cluster variability, is a significant strength. The use of new sampling weights, recently proposed by Elkasabi et al. [71] in the DHS methodology series provides accurate data with lower standard errors. The composite variable of ANC quality significantly contributes to the study methodology. Most studies have paid little attention to the quality of the ANC variable, which was calculated from the content of ANC, but it is an independent predictor of maternal healthcare. The strength of this study emanates from the community’s community’s average distance to a health facility. Hence, calculating the average distance to a health facility provides an approximate distance that helps correct the displaced coordinates for the enumerated areas. However, this study had limitations; one of them is that, due to the retrospective nature, this study has some potential for recall bias, and the answers may not be verifiable due to the secondary nature of the data. Other study limitations include missing values and coordinates that cannot be confirmed due to the secondary nature of the data.

## Conclusions

The findings show that postnatal care service utilization is low and has remained a significant issue in Uganda. This study underscores the negative influence of individual and community-level factors on the utilization of postnatal care services among women in Uganda. The individual-level factors included age (<=19), women of higher parity (2-3, 4+), women with primary and no education, women who reside in rural areas, belonging to the poor bracket of the wealth index, being unemployed, receiving low content of antenatal care services and all the factors had a negative effect in the utilization of postnatal care services. Meanwhile, at the community level, factors such as low media saturation influenced the utilization of postnatal care services among women in Uganda. Furthermore, the study established that women in the Western region were less likely to utilize postnatal care services than other regions. The government of Uganda and other stakeholders must continue to reinforce policies that target adolescent women to utilize maternal health services and also encourage women of high parity to utilize maternal healthcare services. Employment creation to eradicate poverty among women who reside in rural areas and urban poor will go a long way in alleviating suffering. There is a need for continuous monitoring of maternal healthcare programmes across the health facilities regarding the quality of maternal healthcare services offered to pregnant women in Uganda. Programmes that promote access to media sources among communities across Uganda are encouraged. Community education regarding the importance of maternal healthcare services in Western Uganda could help improve reproductive health among women in this region.

## Data Availability

The dataset can be accessed through this website: https://dhsprogram.com/ data/dataset_admin/login_main.cfm?CFID=6055127&CFTOKEN=416a39e1e52181a9-CCE2DAA5-A212-565C-40BD6F8E8C8E5041. Registration is required. This study used the UGIR70FL (Individual Recode –Women with completed interviews – Uganda, 2016).

## Abbreviations

ANC: Antenatal Care
EA: Enumerated Areas
HSDP: Health Sector Development Plan
ICC: Interclass correlation
OR: Odd Ratio
PNC: Postnatal Care
UDHS: Uganda Demographic and Health Survey
WHO: World Health Organization
AOR: Adjusted Odd Ratio
